# Early Enteral Feeding Improves Tolerance of Parenteral Nutrition in Preterm Newborns

**DOI:** 10.3390/nu13113886

**Published:** 2021-10-29

**Authors:** Giovanni Boscarino, Maria Giulia Conti, Maria Di Chiara, Marco Bianchi, Elisa Onestà, Francesca Faccioli, Giorgia Deli, Paola Repole, Salvatore Oliva, Francesco Cresi, Gianluca Terrin

**Affiliations:** 1 Department of Maternal and Child Health, Policlinico Umberto I Hospital, Sapienza University of Rome, 00161 Rome, Italy; giovanni.boscarino@yahoo.com (G.B.); mariagiulia.conti@uniroma1.it (M.G.C.); maria.dichiara@uniroma1.it (M.D.C.); marbi1996@gmail.com (M.B.); elisa.onesta@gmail.com (E.O.); francesca.faccioli@hotmail.it (F.F.); giorgia.deli7@gmail.com (G.D.); paolarepole@gmail.com (P.R.); salvatore.oliva@uniroma1.it (S.O.); 2 Department of Molecular Medicine, Sapienza University of Rome, 00185 Rome, Italy; 3 Neonatal Pathology and Neonatal Intensive Care Unit, Sant’ Anna Hospital, City of Health and Science University Hospital of Turin, University of Turin, 10126 Turin, Italy; francesco.cresi@unito.it

**Keywords:** hyperglycemia, hypertriglyceridemia, metabolic acidosis, very low birth weight (VLBW), enteral nutrition, critical condition, incretin, gut, neonatology, feeding intolerance, trophic feeding, necrotizing enterocolitis

## Abstract

(1) Background: The tolerance of preterm newborns for the high nutritional intakes given by parenteral nutrition (PN) is still debated because of the risk of metabolic complications. Despite enteral nutrition (EN) being the preferred route of nutrition, an exclusive enteral feeding is not always possible, as in preterm newborns, the gut is immature and less tolerant of EN. We aimed to study the impact of a minimal enteral feeding (MEF) on the possible early metabolic complications of PN in a cohort of preterms with gestational age at birth GA ≤ 29 + 6/7 weeks of postmenstrual age. (2) Methods: We divided the study sample in two cohorts: 1) Late-Feeding (cohort 1), newborns who received MEF starting from the 8th day of age, and (2) Early-Feeding (cohort 2), newborns who received MEF, consisting of the administration of at least 4–5 mL/kg/day by the enteral route, in the first 7 days of age. The primary outcome of the study was the rate of at least one metabolic complication, including hyperglycemia, hypertriglyceridemia, or metabolic acidosis. (3) Results: We enrolled 80 newborns (Late-Feeding cohort 51 vs. Early-Feeding cohort 29). The rate of all metabolic complications was statistically higher in the Late-Feeding cohort compared to the Early-Feeding cohort. Binary logistic regression analysis showed that late administration of MEF negatively influenced the rate of all metabolic complications. (4) Conclusions: Early minimal administration of EN is associated with less frequent PN-related metabolic side effects and a higher rate of survival in critically ill newborns.

## 1. Introduction

Current guidelines for preterm neonates recommend high nutritional intakes in order to limit extrauterine growth restriction (EUGR) and the possible long-term consequences of malnutrition [1,2]. During the first postnatal week, when para-physiological intestinal insufficiency limits the use of enteral nutrition (EN), parenteral nutrition (PN) is essential to meet nutritional needs in this vulnerable population [1,2,3]. However, the tolerance of critically ill patients for PN is still debated [4,5,6,7,8,9]. In adults and children observed in an intensive care unit (ICU), a nutritional approach based on a high macronutrient intake significantly affects morbidity and mortality [10,11,12]. Recent evidence reported a higher risk of metabolic complications in newborns receiving enhanced PN [8].

For preterm babies, it is not always feasible to reach a full enteral feeding (FEF) (~120 kcal/kg/day by enteral route) [13] during their first days of age because of their critical clinical conditions. The lack of intraluminal nutrients, such as that observed in subjects receiving total PN (TPN), has deleterious effects on functions of the gastrointestinal tract [3]. The negative consequences of starvation can be countered by EN for a gut virgin to any feeding, and even a small volume of enteral feeding has several advantages when compared with TPN [3,9]. A minimal amount of enteral feeding (defined as minimal enteral feeding, MEF) has trophic effects on intestinal mucosa and improves clinical outcomes of preterm newborns, including a reduced risk of sepsis without an increased occurrence of necrotizing enterocolitis (NEC), and it is a potent stimulus for intestinal development [3,14]. Recent studies have suggested that PN-related complications may be linked, at least in part, to an inactivation of the gut–brain hormonal axis, which is involved in a multitude of physiological processes, including the regulation of glucose and fat metabolism as well as insulin secretion and sensitivity [15,16]. We hypothesized that the administration of a lower amount of EN (defined as MEF) [17] in the first postnatal week could reduce the occurrence of PN-related metabolic complications. We designed a prospective observational cohort study to evaluate the PN tolerance in 2 cohorts of newborns born before 29 + 6/7 weeks of gestational age (GA) receiving MEF or nil per os during the first postnatal week.

## 2. Materials and Methods

### 2.1. Study Design and Population

Starting from 1 January 2013, we prospectively collected, in a specific database, data regarding nutritional intakes, occurrence of hyperglycemia (HG), hypertriglyceridemia (HiTG), metabolic acidosis, morbidity and survival during hospitalization of all newborns with GA ≤ 29 + 6/7 weeks of postmenstrual age (PMA) or birth weight < 1500 g, consecutively observed in the neonatal ICU (NICU) of Policlinico Umberto I.

From 1 July 2017, we changed the nutritional protocol regarding the timing of EN introduction. Before this change in practice, we administered EN only after the first postnatal week, while the new protocol consisted of the introduction of EN in the first week of age. Thus, to verify the effects of the change in nutritional strategy on the occurrence of HG, HiTG and metabolic acidosis related to PN, we designed a study protocol comparing 2 temporal cohorts: Late-Feeding (cohort 1) vs. Early-Feeding (cohort 2).

We considered eligible for the study newborns with GA ≤ 29 + 6/7 weeks of PMA. Newborns with congenital intestinal and extra-intestinal malformation or congenital infections, inborn errors of metabolism and hospital discharge or death within 72 h after birth in critical conditions were excluded [18,19,20,21,22,23].

We calculated a minimum sample size of 116 patients (2-sample *t*-test, 80% of power in hypothesis test, 0.05 of type 1 error, 2-tailed test, drop out 15%) to demonstrate a difference of about 25% in the rate of occurrence of at least one metabolic complication (i.e., HG, HiTG or metabolic acidosis) between the two study cohorts. Thus, we recruited subjects of cohort 1 back in time until the estimated sample size was reached. Subjects of cohort 2 were enrolled prospectively starting from July 2017. Considering this change in our clinical practice, we scheduled an interim analysis after the enrollment of 50% of patients in cohort 2.

### 2.2. Nutritional Protocol

EN was started in both study cohorts as MEF. We administered MEF at a volume ranging from 4–5 mL/kg (1–2 bolus day) to 10–20 mL/kg (6–8 bolus day), according to the signs of feeding tolerance. In particular, newborns without signs of feeding intolerance received 10–20 mL/kg/day, while patients that showed mild feeding intolerance (i.e., abdominal distention and/or gastric residuals not blood/bile stained) received 4–5 mL/kg/day. In subjects presenting the alarm signs of feeding intolerance that suggest NEC (erythematic abdominal wall, absence of bowel sounds or blood in the stools or in aspirates associated with radiological marker), MEF was not started until the alarm signs of NEC completely disappeared.

EN was increased in both cohorts in the absence of signs of feeding intolerance by 10–20 mL/kg daily starting from the 8th day of age. If newborns presented signs of mild feeding intolerance, we administered MEF at a volume of 4–5 mL/kg/day for at least 24 h. When we observed the alarm signs of feeding intolerance that suggest the early stage of NEC, EN was suspended for at least 24 h. We restarted EN when signs of feeding intolerance completely disappeared. During refeeding, we administered MEF as described above.

We used a PN protocol in line with the current European guidelines for PN during the study period (Table S1) [2,24]. Macronutrients administered by PN were calculated based on the published manufacturer’s labels, including proteins (TrophAmine 6% Braun Medical Inc., Irvine, CA, USA), lipids (Smoflipid, Fresenius Kabi, Lake Zurich, IL, USA) and carbohydrates (Dextrose injection 10%, Fresenius Kabi, USA) expressed in g/kg/day. The mothers’ own milk was administered when available, while donor milk was not available during the study period. Preterm formula (Pre-Nidina Nestlè, Milan, Italy) was administered when human milk was not available or sufficient. Preterm human milk was assumed to contain 65 Kcal/100 mL (1.5 g of protein/100 mL, 3.5 g of fat/100 mL, 6.9 g of carbohydrate/100 mL) [25].

### 2.3. Outcome

The primary outcome of the study was the rate of metabolic complications. We defined the presence of at least one metabolic complication as the occurrence of HG or HiTG or metabolic acidosis. Blood glucose levels were monitored by the validated micro-method four to eight times a day, according to clinical conditions, through capillary blood sampling [26]. We defined HG as the presence of two consecutive blood glucose levels greater than 180 mg/dL, at least 3 h apart. We measured the serum triglycerides every 72 h in newborns receiving PN, and we considered the level of plasma triglycerides greater than 150 mg/dL as HiTG. Blood gas analysis was performed at least once daily during the first postnatal week; metabolic acidosis was defined as base excess <10 mmol or pH < 7.25 with pCO2 < 50.

We also compared the rate of survival during hospital stay between the two study cohorts.

### 2.4. Data Collection

Physicians, in charge of the babies and making nutritional corrections in relation to their clinical status, evaluated the eligibility criteria unaware of the study aims. Researchers not involved in clinical practice and blinded for the study aims recorded neonatal, nutritional, and metabolic complications data for statistical analysis in a customized database, as previously stated. Specifically, we collected data regarding intrauterine growth restriction (IUGR), pregnancy-induced hypertension, antenatal corticosteroids administration (as an intramuscular steroid cycle in two doses of 12 mg over a 24 h period), GA, birth weight, to be small for GA (SGA), sex, type of delivery, twin’s pregnancy, pH at birth, Apgar score at 5 min after birth and respiratory distress syndrome. Data regarding nutritional intake and prematurity-related morbidities (NEC, bronchopulmonary dysplasia, sepsis proven by positive culture, retinopathy of prematurity, periventricular leukomalacia and intraventricular hemorrhage before and after 7 days of age) [27,28,29,30,31] were also collected during the entire hospital stay. 

### 2.5. Statistics

Statistical Package for Social Science software (SPSS Inc., Chicago, IL, USA), version 25.0, was used to perform statistical analysis. We checked for normality using the Shapiro–Wilk test. The mean and standard deviation summarized normally distributed continuous variables, and the number and percentage described categories variables. We used the χ^2^ test for categorical variables and *t*-test or Mann–Whitney for paired and unpaired variables.

A binary regression analysis was performed to evaluate the influence of pH at birth, to be small for gestational age at birth, male sex and the exposure cohort on the rate of rate of metabolic complications. The level of significance for all statistical tests was two-sided (*p* value < 0.05). The data were elaborated by a blinded statistician.

## 3. Results

**Figure 1:**
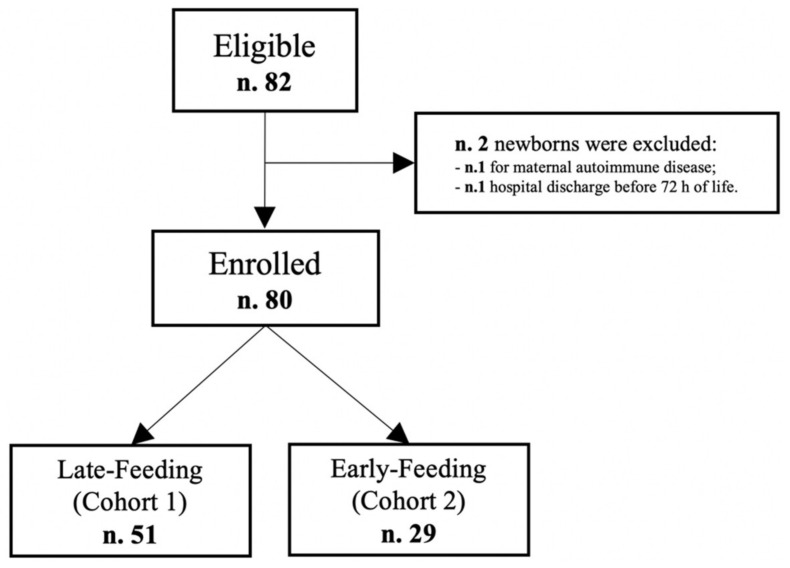
Flowchart of the study sample.

Ad interim analysis performed in December 2019 showed the results discussed below. Of the 82 newborns that met eligible criteria, we enrolled 80 neonates (Figure 1). 

The baseline clinical characteristics of the study sample are described in Table 1. The length of hospital stay was similar between the two cohorts (Late-Feeding 84 ± 55 days vs. Early-Feeding 91 ± 45 days, *p* value 0.600).

**Figure 2:**
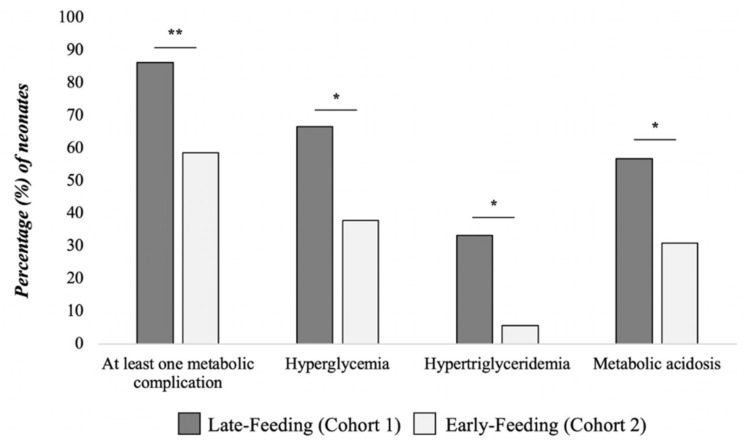
Rate of metabolic complications in the study sample. Notes. * *p* value < 0.05; ** *p* value < 0.01.

Neonates enrolled in the Late-Feeding cohort showed a higher rate of all metabolic complications than that of the Early-Feeding cohort (Figure 2).

The rate of survival during hospital stay was higher in the Early-Feeding cohort than that in the Late-Feeding cohort (Late-Feeding 80.4% vs. Early-Feeding 96.6%, *p* value = 0.040). The two study cohorts showed little difference for prematurity-related morbidity conditions (Table 2). There were also not differences for PN macronutrient intakes in the first weeks of age between the two study cohorts (Table 3). 

**Figure 3:**
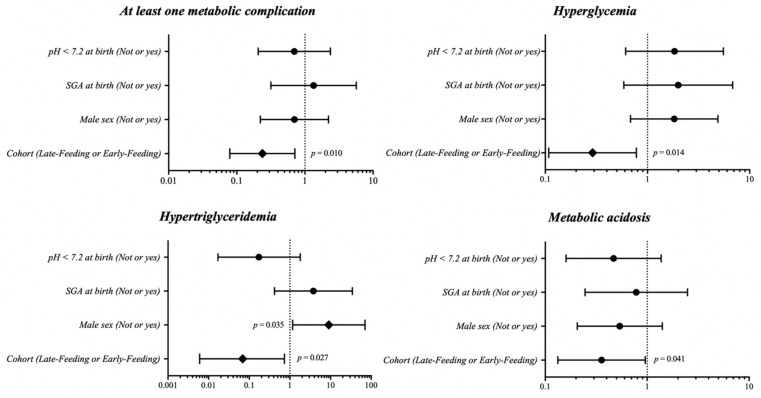
Binary logistic regression analysis to evaluate the influence of covariates on primary outcomes. Notes: SGA—small for gestational age.

In binary logistic regression analysis, the late administration of MEF after the first 7 days of age negatively, independently influenced the rate of HG, metabolic acidosis and at least one metabolic complication (Figure 3). The rate of HiTG was influenced by male sex and late administration of MEF (Figure 3). We performed post hoc analysis with 82.6% of power (0.05 of type 1 error, 2-tailed test), and we decided to suspend the study.

## 4. Discussion

Our study provides compelling evidence that early introduction of enteral feeding, in a sample of preterm neonates born before 29 + 6/7 weeks of PMA with a gut virgin to any feeding, reduces PN-related complications and is associated with an increased rate of survival.

Despite previous studies demonstrating the benefits of MEF for preterm newborns [14,32,33], to the best of our knowledge, this is the first study to evaluate the protective role of MEF on metabolic complications related to PN in early neonatal life. Recent evidence showed the positive role of EN in adults and children in critically ill conditions. The benefit of EN for glucose homeostasis has also been demonstrated in adult populations. Campbell et al. demonstrated that, in an adult population, continuous enteral feeding reduced the risk of hypoglycemia and HG compared to intermittent enteral feeding, with a lesser insulin requirement and more stable glucose control [34]. Prakash et al. [35], in an RCT, demonstrated the effects of early vs. late EN introduction on the mortality rate in children observed in pediatric ICU. They found a statistical trend (early 29.8% vs. late 48.1%) that did not reach statistical significance (*p* value = 0.07) for mortality outcomes. However, this study is underpowered, and metabolic complications were not evaluated in this trial. Srinivasan et al. [36], in a secondary analysis of the Heart and Lung Failure-Pediatric Insulin Titration RCT that enrolled critically ill children observed in pediatric ICU with HG requiring inotropic support and/or invasive mechanical ventilation, demonstrated that early administration of EN improved clinical outcomes, including mortality, length of hospital stay, ventilator-free days and less organ dysfunction. These results were confirmed after adjusting for age, BMI z-score, inotropic support at the time of study randomization, primary reason for ICU admission and severity of illness. They enrolled infants with HG, so they did not evaluate the specific influence of EN on metabolic complications. 

It is possible to speculate that the positive effects we observed in our cohort exposed early to MEF are associated with gut incretin secretion. Despite the benefit of PN, animal and human studies have demonstrated that exclusive TPN leads to intestinal atrophy, reduces gastrointestinal blood flow, creates digestive dysfunction and delays development of the mucosal epithelium and innate immune function [37]. Studies on neonatal pigs showed that removing enteral feeding and maintaining TPN reduced portal venous and superior mesenteric atrial blood flow, with an increase in epithelial cell apoptosis and villus atrophy [38]. As a villus atrophy progresses, there is a reduction in intestinal digestive enzyme activity [39]. It has been demonstrated that intravenous nutrition reduces incretin production compared to EN [40]. Meessen et al. [16] demonstrated that intravenous nutrient administration eliminates the physiological plasma bile acid response. In this study, the low plasma bile acids were associated with an impaired gut hormone response (FGF19, incretins and GLP-2). These alterations could be responsible for PN-associated metabolic side effects. Specifically, the incretin hormone increases insulin and lipase production and increases the potassium excretion through renal function. In the case of enteral feeding administration, the incretin release reduces the level of glucose (because of insulin response), reduces blood fat (because of lipase production) and finally limits the occurrence of metabolic acidosis [40]. All of these physiological mechanisms are inhibited in cases of PN support without minimal EN administration. 

Despite the innovative findings, our study should be interpreted considering several limitations. The association between early MEF and the reduced risk of metabolic complications may be related to the effects of random error or unconsidered confounding factors. In a multivariate model, we corrected these results for confounding variables that could have influenced our primary outcomes. However, variables unknown to or not considered in our statistical model might have influenced the outcomes. This was not an RCT. Individualized PN solution corrections are the milestone of our policy on PN to avoid the deleterious consequences of complications related to the intravenous administration of PN [7,8,26,41]. To reduce this bias, we considered eligible newborns born before 29 + 6/7 weeks of PMA in TPN (more than 85% of total nutrition) of a large cohort of babies. For ethical reasons and because of the risk of NEC, it is not easy to design an RCT evaluating the nutritional support (EN specifically) of neonates in critically ill conditions. Additionally, the risk of a lack of equipoise within physicians caring for preterm infants could be very high. To limit selection bias, physicians evaluating eligibility were blinded to the study aims and used objective inclusion criteria (GA). To limit observer bias, the data for the analysis were collected by researchers not involved in eligibility assessment and who were unaware of cohort assignment. We discussed nutritional practices with physicians and defined a protocol for the collection, measurement and interpretation of data before starting the study. A blinded statistician performed the data analysis. We divided the cohorts on a temporal basis, which also represents a bias in the study. Despite no changes in the care policies during the study period and the similar baseline characteristics of the two study cohorts, it is not possible to exclude the notion that unknown differences in the clinical practice or changes in the caregivers composition may have influenced the results. To reduce the bias related to GA, we enrolled only newborns born before 29 + 6/7 weeks of PMA. We considered the total amount of maternal milk and preterm formula as EN. The preterm formula used for EN did not change during the entire study period. We used a volume of least 4–5 mL/kg/day as MEF. We decided to start with this volume for tolerant newborns on the bases of our preliminary data that revealed a good tolerance of this kind of MEF in comparison with MEF administered with 10–20 mL/kg/day. We cannot exclude that similar results could also be obtained with greater volume of EN.

## 5. Conclusions

In conclusion, the early introduction of enteral feeding could be used as a potential protective nutritional strategy to reduce the metabolic complications of an enhanced PN protocol. Further studies are needed to confirm the effects of early introduction of EN on survival and to evaluate the consequences on long-term neurodevelopment.

## Figures and Tables

**Table 1:** Baseline characteristics of the study sample.

	Late-Feeding (Cohort 1) (*n* = 51)	Early-Feeding (Cohort 2) (*n* = 29)	*p* Value
Intrauterine growth restriction, N. (%)	7 (13.7)	4 (13.8)	0.629
Pregnancy-induced hypertension, N. (%)	12 (23.5)	5 (17.2)	0.481
Antenatal corticosteroids ^a^, N. (%)	30 (58.8)	18 (62.1)	0.856
Gestational age, weeks	27 ± 2	27 ± 2	0.086
Birth weight, g	864 ± 258	952 ± 232	0.099
Small for gestational age at birth, N. (%)	11 (21.6)	5 (17.2)	0.612
Male sex, N. (%)	27 (52.9)	18 (62.1)	0.429
Cesarean section, N. (%)	40 (78.4)	25 (86.2)	0.392
Twins, N. (%)	12 (23.5)	6 (20.7)	0.770
pH at birth	7.2 ± 0.1	7.2 ± 0.1	0.892
5 min Apgar score, mean (IQR)	7 (3)	7 (1)	0.055
Respiratory distress syndrome, N. (%)	45 (88.2)	28 (96.6)	0.278

^a^ Intramuscular steroid cycle in two doses of 12 mg over a 24 h period. Data are shown as mean ± standard deviation when not specified.

**Table 2:** Morbidity during hospital stay of the study sample.

	Late-Feeding (Cohort 1) (*n* = 51)	Early-Feeding (Cohort 2) (*n* = 29)	*p* Value
Necrotizing enterocolitis	0 (0)	1 (3.4)	0.362
Bronchopulmonary dysplasia	10 (19.6)	2 (6.9)	0.098
Sepsis proven by positive culture	9 (17.6)	5 (17.2)	0.963
Retinopathy of prematurity	20 (39.2)	14 (48.3)	0.431
Periventricular leukomalacia	1 (2.0)	1 (3.4)	0.597
Intraventricular hemorrhage:			
Before 7 days of age	9 (17.6)	1 (3.4)	0.058
After 7 days of age	4 (7.8)	0 (0)	0.153

Data are shown as number (percentage).

**Table 3:** Parenteral nutrition macronutrient intake of the study sample.

	Late-Feeding (Cohort 1) (*n* = 51)	Early-Feeding (Cohort 2) (*n* = 29)	*p* Value
Energy intake, kcal/kg/week	626.4 ± 191.5	635.9 ± 186.6	0.885
Protein intake, g/kg/week	24.3 ± 8.4	23.3 ± 7.3	0.635
Glucose intake, g/kg/week	84.8 ± 25.6	84.2 ± 23.3	0.885
Fat intake, g/kg/week	19.9 ± 6.3	21.1 ± 6.8	0.548

Data are shown as mean ± standard deviation.

## Data Availability

Data are available upon reasonable request. All data relevant to the study are included in the article. Access to raw data will be provided upon request.

## Supplementary Materials

The following are available online at https://www.mdpi.com/article/10.3390/nu13113886/s1, Table S1: Parenteral nutrition protocol.
