# Supplementary material for: Early Enteral Feeding Improves Tolerance of Parenteral Nutrition in Preterm Newborns
Source: Nutrients. 2021 Oct 29;13(11):3886. doi: 10.3390/nu13113886 (PMC8621891; doi:10.3390/nu13113886)
Supplement: Supplementary file 1 [file nutrients-13-03886-s001.zip › nutrients-1407198-supplementary.pdf]

Table S1. Parenteral nutrition protocol.

	Birth weight < 1000 g	Birth weight ≥ 1000 g
**Energy** *(kcal/kg/day)*		
Starting dose	45	45
At day of life	0	0
Target dose	105	100
At day of life	7	7
**Proteins** *(g/kg/day)*		
Starting dose	2.0	2.0
At day of life	0	0
Target dose	4.0	3.5
At day of life	7	7
**Dextrose** *(g/kg/day)*		
Starting dose	7.0	7.0
At day of life	0	0
Target dose	14.0	14.5
At day of life	7	7
**Lipids** *(g/kg/day)*		
Starting dose	1.0	1.0
At day of life	0	0
Target dose	3.5	3.0
At day of life	7	7
